# Supplementary material for: Molecular Imaging and Quantification of Smooth Muscle Cell and Aortic Tissue Calcification In Vitro and Ex Vivo with a Fluorescent Hydroxyapatite-Specific Probe
Source: Biomedicines. 2022 Sep 14;10(9):2271. doi: 10.3390/biomedicines10092271 (PMC9496085; doi:10.3390/biomedicines10092271)
Supplement: Supplementary file 1 [file biomedicines-10-02271-s001.zip › Figure S1.pdf]

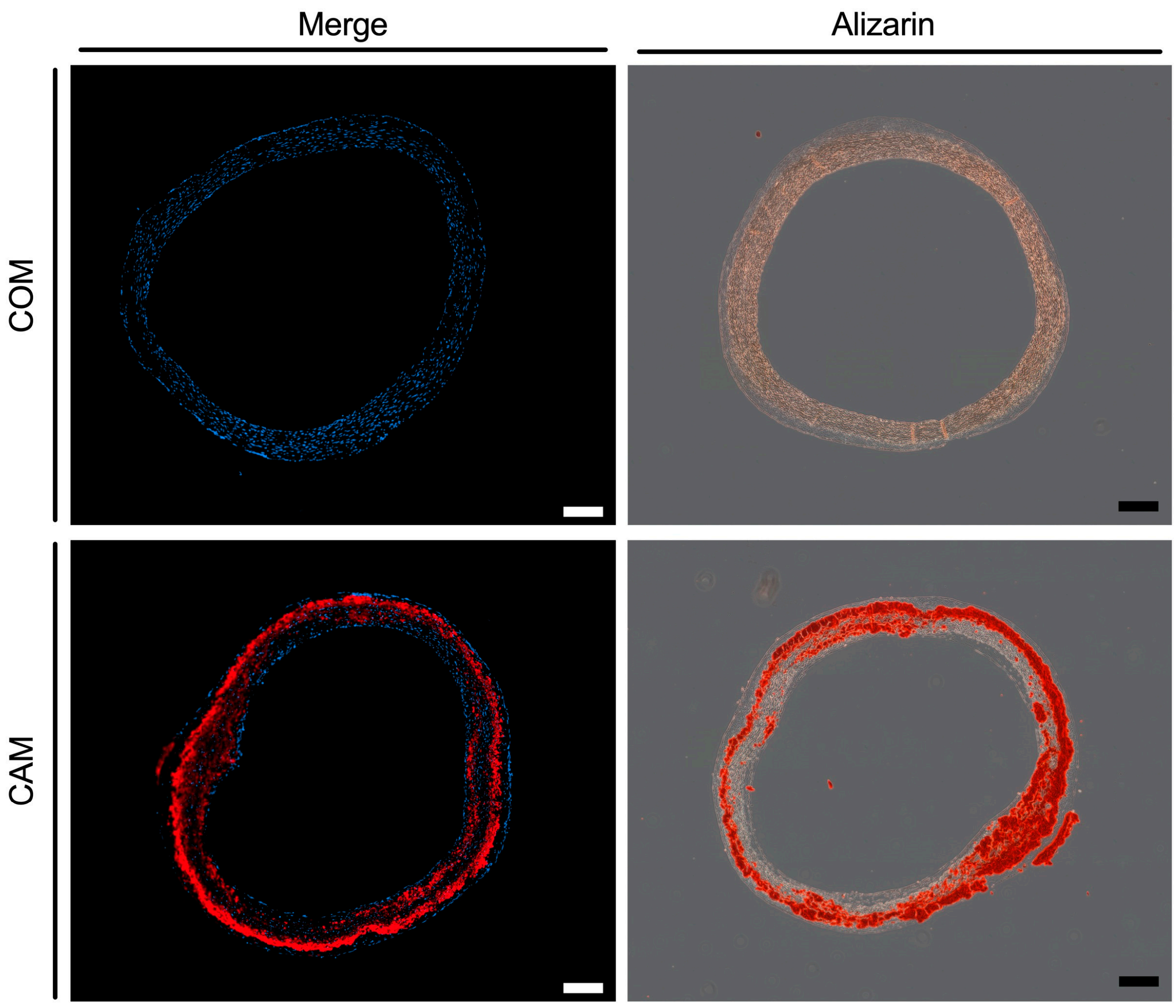

Figure S1. Representative images of aortic staining for hydroxyapatite with OsteoSense™680EX (OSTM) (red channel) and cell cores with Hoechst33432 (blue channel) or Alizarin Red upon stimulation with control medium (COM) or calcification medium (CAM), respectively, for 14 d.
